# Prefrontal Dopamine in Flexible Adaptation to Environmental Changes: A Game for Two Players

**DOI:** 10.3390/biomedicines11123189

**Published:** 2023-11-30

**Authors:** Emanuele Claudio Latagliata, Cristina Orsini, Simona Cabib, Francesca Biagioni, Francesco Fornai, Stefano Puglisi-Allegra

**Affiliations:** 1 I.R.C.C.S. Fondazione Santa Lucia, 00143 Rome, Italy; cristina.orsini@uniroma1.it (C.O.); simona.cabib@uniroma1.it (S.C.); 2 Department of Psychology, Sapienza University of Rome, 00185 Rome, Italy; 3 I.R.C.C.S. Neuromed, Via Atinense 18, 86077 Pozzilli, Italy; francesca.biagioni@neuromed.it (F.B.); francesco.fornai@neuromed.it (F.F.); 4 Department of Translational Research and New Technologies on Medicine and Surgery, University of Pisa, 56126 Pisa, Italy

**Keywords:** medial prefrontal cortex, nucleus accumbens, dopamine, extinction, goal-directed, therapy

## Abstract

Deficits in cognitive flexibility have been characterized in affective, anxiety, and neurodegenerative disorders. This paper reviews data, mainly from studies on animal models, that support the existence of a cortical–striatal brain circuit modulated by dopamine (DA), playing a major role in cognitive/behavioral flexibility. Moreover, we reviewed clinical findings supporting misfunctioning of this circuit in Parkinson’s disease that could be responsible for some important non-motoric symptoms. The reviewed findings point to a role of catecholaminergic transmission in the medial prefrontal cortex (mpFC) in modulating DA’s availability in the nucleus accumbens (NAc), as well as a role of NAc DA in modulating the motivational value of natural and conditioned stimuli. The review section is accompanied by a preliminary experiment aimed at testing weather the extinction of a simple Pavlovian association fosters increased DA transmission in the mpFC and inhibition of DA transmission in the NAc.

## 1. Introduction

The ability to adapt to new, changing, or unplanned events is known as cognitive or behavioral flexibility [1]. Deficits in cognitive flexibility have been characterized in psychiatric and neurological diseases [2,3,4]. The process is well modeled in experimental conditions by the acquisition and extinction of associative learning in humans and non-human animals. Indeed, through associative training, a neutral stimulus (conditioned stimulus, CS) acquires the value of an associated aversive or rewarding stimulus (unconditioned stimulus, US). Consequently, the CS becomes capable of orienting and controlling behavior. In extinction, the repeated experience of the CS in the absence of the US progressively reduces its control over behavior, allowing disengagement from previously pursued strategies. Therefore, brain mechanisms mediating associative learning play a major role in engaging and disengaging the organisms from pursuing specific behavioral strategies.

The possibility of studying associative learning in different species offers the rare opportunity to fully translate sophisticated neuroscientific findings collected in animal models into clinical research. One major finding obtained through animal research is the role of dopamine (DA) neurons of the mesencephalon in the modulation of CS values. Indeed, mesencephalic DA neurons of primates are activated by the US before associative training, but following US–CS association they are only activated by the CS [5]. This finding strongly contributed to the view of DA transmission as the mediator of associative learning, although the complexity of DA transmission goes well beyond phasic activation of unspecified DA neurons [6]. Indeed, mesencephalic DA neurons send their terminals to functionally different brain areas, their phasic and tonic activity contributing to local transmission on different timescales, and a complex circuital organization modulates this transmission [7,8].

Thus, activation of DA neurons of the ventral tegmental area (VTA), the mesencephalic location of neurons specifically targeting the limbic brain circuit, is both necessary and sufficient to reduce behavioral control by an aversive CS (see [9] for a review). This outcome was attributed to the rewarding effects of safety experienced when CSs cease to be followed by the aversive USs [9]. However, aversive stimuli also activate VTA DA neurons, although these neurons target different limbic areas [8]. Finally, evidence collected in human subjects supports the role of DA transmission in the extinction of aversive CSs through the specific engagement of the meso-cortical-striatal circuitry [10,11].

In mammals, action is the outcome of a brain system that connects the cortex with subcortical areas through a series of parallel loops that are modulated by mesencephalic DA, hence the definition of the mesocortical–striatal circuitry. Indeed, the striatum is the main subcortical node of this circuit, and it receives and sends back information from and to the motor and limbic cortical areas. It also receives DA from the VTA and substantia nigra (SN) compartments of the mesencephalon. 

Since cortical–striatal loops are parallel, they segregate into two distinct circuits: (1) a motor circuit, made up of motor cortices, the dorsal striatum, and the SN; and (2) the limbic circuit, responsible for the emotional–motivational drives that direct action toward specific goals [12,13]. The latter includes targets of VTA DA neurons involved in associative learning, such as the medial prefrontal cortex (mpFC), the limbic striatum (nucleus accumbens: NAc), the amygdala, and the hippocampus. The amygdala is engaged in the acquisition of the association between US and CS while the hippocampus is required for the association of configurations of stimuli, such as spatial configurations or timelines. The NAc is the mediator of behavioral responses to CSs and USs, and of their hedonic sign (i.e., negative or positive). There is compelling evidence that the NAc’s DA response is transferred from USs to CSs by associative training [14,15]. Finally, the mpFC mediates both the acquisition and extinction of associative learning [16,17,18,19,20,21,22]. 

The aim of this paper is to present evidence supporting the hypothesis that DA transmission in the limbic cortical–striatal circuit mediates cognitive/behavioral flexibility. To this aim, the remainder of this paper is organized as a descriptive review of published data supporting this hypothesis. Moreover, we shall present preliminary findings supporting opposite changes in tonic DA levels within the mpFC and NAc when rats cease to express an appetitive response toward amphetamine-associated stimuli, consistent with our hypothesis. 

## 2. The Cortical–Striatal DA Transmission in Health and Disease

**Figure 1:**
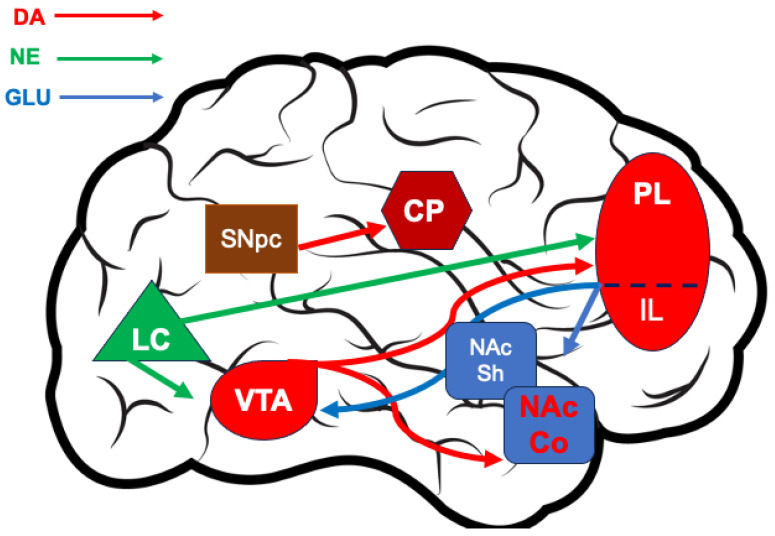
Schematic representation of the main brain areas and connections of interest in this review: Dopaminergic neurons from the ventral tegmental area (VTA) project to the medial prefrontal cortex (mpFC)’s prelimbic (PL) and infralimbic (IL) subregions, along with the nucleus accumbens shell and core (NAcSh and NAcCo, respectively), all involved in specific ways in cognition, emotion, and motivation finalized to adaptive behavior. Dopamine projections from the substantia nigra pars compacta (SNpc) to the striatum (caudate–putamen, CP) are involved in voluntary movements, and their crucial role in Parkinson’s disease (PD) is well known. Moreover, the nigrostriatal system is crucial in behavioral disorders characterized by loss of flexibility, as well as in compulsive behavior characterizing neurodegenerative (PD) and neuropsychiatric disorders. Dopaminergic neurons in these brainstem regions receive direct and indirect excitatory glutamatergic afferents from the prefrontal cortex. Norepinephrinergic neurons in the locus coeruleus (LC) project to the mpFC where, synergically with dopamine, they modulate dopamine transmission in the accumbens, forming the prefrontal/accumbal catecholamine system [26]. The LC modulates the VTA, thus influencing dopamine transmission. DA: dopamine. NE: norepinephrine. GLU: glutamate.

As discussed, a complex circuitry regulates DA transmission in terminal targets (Figure 1). Studies in animal models indicate that mpFC DA transmission can modulate mesoaccumbens DA release. Indeed, tonic extracellular levels of DA in the NAc shell are modulated by excitatory input of mpFC–NAc projection neurons [23,24]. Moreover, a recent study using ex vivo brain slices found that mpFC layer 5 neurons projecting to the NAc were inhibited by the activation of local VTA DA terminals [25]. On the other hand, norepinephrine (NE) transmission in the mpFC is required for the increase in DA availability in the NAc elicited by aversive or rewarding USs, and for US–CS association. Indeed, neurotoxic depletion of NE in the mpFC prevents the US-induced increase in tonic DA in the NAc, as well as the US–CS association [26]. These findings support the conclusion that CSs acquire the ability to increase DA’s availability in the NAc through association with negative or positive USs.

In novel aversive experiences, tonic DA levels in the NAc are increased to support the expression of species-typical and previously acquired defensive strategies. This DA response by the NAc is driven by NE transmission in the mpFC, because neurotoxic depletion of NE in the mpFC prevents it. Both NE increases in the mpFC and DA increases in the NAc cease when the animal appraises that these defensive strategies are ineffective, i.e., that the aversive stimuli cannot be escaped or removed. Indeed, in this case, the NAc’s DA levels are reduced below those observable at a resting state. This response is driven by increased DA transmission in the mpFC and supports the expression of conservative passive coping strategies. Indeed, neurotoxic depletion of DA in the mpFC or local blockade of DA receptors can prevent the inhibition of DA in the NAc and recover the expression of active coping strategies [27]. These findings show that enhanced DA transmission in the mpFC can inhibit the availability of DA in the NAc and, consequently, disengage organisms from ongoing behavioral responses. Moreover, they indicate that local catecholaminergic transmission in the mpFC controls DA’s availability in the NAc in associative and non-associative settings. Interestingly, dysfunctional connectivity between the mpFC and NAc is considered to be a core feature of different disturbances, such as addiction, schizophrenia, depression, and Parkinson’s disease [4,28,29,30,31,32,33,34]. Moreover, findings from human studies indicate striatal DA transmission to play a paramount role in keeping the cortical–striatal–thalamic–cortical connectivity functional [29,30,31,35,36,37]. A progressive depletion of striatal DA is the hallmark of Parkinson’s disease (PD). This condition is the outcome of the degeneration of DA neurons located in the SN and is responsible for the characteristic motor symptoms. However, there is strong evidence that reduced DA availability in the NAc precedes typical motor deficits in PD, and 30% of PD patients present non-motor deficits long before the appearance of classic motor symptoms [38,39]. Several studies have found reduced connectivity between the anterior cingulate cortex (ACC; a component of the mpFC) and the NAc in PD [31]. Remarkably, substantial evidence indicates that the human ACC and rodents’ prelimbic cortex (PL) play the same role in supporting threat responses and connect with the same limbic/striatal areas [40]. The influence of mpFC DA on humans’ functional cortical–striatal connectivity has been much less explored. Nonetheless, different indices of mesocortical DA dysfunction have been observed in PD patients showing pathological gambling, depression, or freezing of gait, as well as in bipolar disorder [32,41,42,43].

The findings of studies on rodents subjected to experimental DA depletion by the neurotoxin 6-OHDA and imaging studies of PD patients converge in suggesting a causal relationship between a hypodopaminergic state, chronic pain, and acute hyperalgesia. This conclusion is supported by the observation that chronic pain in PD patients is susceptible to treatment with dopaminomimetic drugs such L-DOPA or DAD2 receptor (D2R) agonists [44,45]. On the other hand, recent results indicate a crucial role of the locus coeruleus (LC), the main source of NE in the brain, in pain and stress-related responses [46], as well as indicating that complex interactions among brainstem pathways and NE receptors modulate both the inhibition and facilitation of pain [47]. Several studies have addressed the potential involvement of the LC in the pathogenesis of neurodegenerative disorders [48], and the development of MRI-based methods aimed at visualizing the LC has allowed for the evaluation of its integrity in vivo. In PD, the LC is one of the first areas to undergo degeneration. Thus, reduced DA availability in the mesoaccumbens in stressful conditions, fostered by impaired mpFC NE responsivity, could be one source of chronic pain in PD. In addition, the NE system exerts anti-inflammatory and neuroprotective effects on the VTA’s DA neurons, protecting them from degeneration; consequently, it is highly probable that NE damage in the LC will affect the disease progression and the severity of DA hyper-responsivity in the NAc [49,50,51,52,53,54]. 

Together, findings obtained from animal models indicate that variations in catecholamine levels in the mpFC control flexible behavioral adaptations to novel aversive environments, as well as the association between CSs and either aversive or rewarding USs, by modulating NAc DA transmission. Moreover, clinical data support the role of altered DA transmission in the limbic cortical–striatal circuit in the expression of non-motor symptoms.

## 3. Cognitive/Behavioral Flexibility, Cortical–Striatal DA, and Goal Value

Impaired flexibility is a known cognitive deficit associated with PD, but also with the effects of DA-targeted treatment in PD [2,3,51,52]. In humans and animal models, this phenotype is measured by reversal learning and set-shifting tests, which require the acquisition and extinction of associative learning. 

Indeed, reversal learning measures the ability to disengage from ongoing behavior to acquire a new strategy. Results obtained for reversal learning in animal models demonstrate that the two processes are sequentially engaged in two different phases. The first phase involves the inhibition of a previously acquired response, while the second involves the acquisition of a new one. It has also been demonstrated that the first phase is facilitated by the selective blockade of DA transmission in the NAc [53,54], and by enhanced DA transmission in the mpFC [55]. Instead, DA transmission in the dorsal striatum is required for the acquisition of a new operant strategy in the second phase of reversal learning [53,54]. Interestingly, stable acquisition of a passive coping strategy in an inescapable aversive situation also requires DA transmission in the dorsal striatum, although the initial inhibition of ineffective active coping strategies requires the inhibition of DA in the NAc by enhanced DA in the mpFC (see [27] for a review).

As previously discussed, both the mpFC and NAc receive DA from neurons located in the VTA, while the dorsal striatum receives DA from neurons located in the SN. The SN’s DA neurons modulate the circuit supporting habitual responses (i.e., responses automatically elicited by CSs) [56,57], while the VTA’s DA neurons modulate circuits involved in goal-directed actions [58,59]. The two circuits are active in parallel during the learning of a new association, but they compete to control behavior in the presence of the acquired CS. Finally, competition for behavioral control between the two circuits engages local mesencephalic and striatal–mesencephalic circuits capable of shifting the activation of DA neurons from one population to the other [31,57,60,61]. Habitual responses mediated by DA projections from the SN to the dorsal striatum are needed for daily activities that can be performed automatically (such as walking or driving a car). Nonetheless, a circuital imbalance favoring automatic responses over goal-directed actions fosters behavioral disturbances characterized by perseveration, such as pharmacological or behavioral dependencies, obsessive–compulsive disorder, and some non-motor symptoms in PD [31,62,63].

In contrast with habitual responses, goal-directed actions are flexible and controlled by the motivational salience of the goal. Behavior is also controlled by the valence (positive vs. negative) of goals, which defines the direction of the behavior elicited (approach, search, consume vs. escape, avoid) and is computed by a complex circuit that does not include DA transmission [64,65]. There is compelling evidence, instead, that DA transmission in the NAc is required for stimuli to be perceived as salient, regardless of their valence [64,66,67], as also shown by neuroimaging in humans [68]. USs’ incentive salience is modulated by physiological states such as hunger [69], and CSs acquire incentive salience through association with USs, together with USs’ ability to elicit DA responses in the NAc [14,15]. Thus, impaired DA transmission in the NAc could devaluate an unconditioned goal or a previously acquired CS [67,68], leading to the suppression of well-established avoidance/escape or search/pursue strategies. 

The reviewed findings on cognitive behavioral flexibility suggest the following conclusions: (1) reduced DA transmission in the NAc and enhanced DA in the mpFC facilitate the extinction of a previously acquired behavioral strategy in reversal learning; (2) DA in the dorsal striatum mediates the acquisition of alternative behavioral responses in associative and non-associative learning; (3) DA transmission in the NAc is required for USs and CSs to be perceived as salient and to be able to direct and support goal-directed behaviors.

## 4. Updating Goal Value

The reviewed findings allow for a number of conclusions: (1) DA transmission in the mpFC and NAc plays important roles in flexible behavioral adaptation to variable conditions; (2) a specific cortical–striatal catecholaminergic circuit is engaged by this process; (3) NE transmission in the mpFC enhances DA release in the NAc in the presence of salient USs and is required for US–CS associations; (4) in novel aversive situations, DA in the mpFC controls tonic DA release in the NAc and the expression of appropriate coping strategies; (5) both enhanced DA transmission in the mpFC and reduced DA transmission in the NAc are involved in cognitive/behavioral flexibility and mediate disengagement from previously acquired behaviors. 

On the other hand, whereas the role of NE in the mpFC in the acquisition of a CS–US association through the stimulation of DA release in the NAc has been experimentally demonstrated, the role of DA in the mpFC in the extinction of a CS–US association through the inhibition of DA release in the NAc remains to be tested. Moreover, as discussed, the activation of DA neurons in the VTA by the extinction of an aversive US–CS association is interpreted as the outcome of relief [9], a positive state that should depend on enhanced DA release in the NAc, while the engagement of DA transmission in the extinction of either positive or negative CSs is presumed rather than demonstrated [70]. The extinction of rewarding associations has mostly been evaluated in operant/instrumental protocols; however, we have recently reported that a temporary blockade of NE by the α1-adrenoreceptor antagonist prazosin in the PL by local prazosin infusion anticipates extinction of the preference for a compartment associated with the effects of amphetamine (AMPH) in conditioned place preference (CPP) [71]. As discussed, NE transmission in the mpFC enhances DA availability in the NAc; therefore, the blockade of NE transmission in the PL could prevent CS-elicited DA release in the NAc, hence its attractiveness. Indeed, in the same paper, mice showing anticipated extinction of AMPH-CPP due to the blockade of NE transmission in the PL also showed markers of plasticity in the NAcCo [71]. 

Therefore, we decided to evaluate whether the extinction of AMPH-CPP was accompanied by enhanced release of tonic DA in the PL and inhibition of tonic DA release in the NAcCo, as the preliminary experiment of a study on the role of mpFC DA’s control over NAc DA in the extinction of a rewarding Pavlovian association, using the same protocol employed in previous experiments [71].

Indeed, we used a paradigm of spatial conditioned place preference (CPP) in rats to compare the effects of daily short (20 min) or long (80 min) exposure to a context previously associated with the effects of AMPH on the development of a stable extinction of the conditioned preference. 

The nucleus accumbens core (NAcCo) subregion plays a crucial role in the extinction of salient stimulus-related CSs [57,72,73,74,75,76,77,78,79]. However, there is no evidence that a successful extinction is also associated with the inhibition of DA release in the mesoaccumbens.

Thus, we evaluated DA release by intracerebral microdialysis, aimed at assessing the time-course of DA during re-exposure to the learning context within the PL and NAcCo. 

The experimental methods and detailed results can be found in the Supplementary Materials. Here, we summarize the main findings of these experiments: (1) longer extinction sessions (80 min vs. 20 min) were required for an effective extinction of preference for the compartment associated with the effects of amphetamine; (2) eighty minutes of exposure to the AMPH-paired compartment, in the absence of drug effects, was required to elicit a significant increase in tonic DA in the PL; (3) a significant reduction in tonic DA outflow in the NAcCo was only observable after 80 min of exposure to the AMPH- paired compartment in the absence of drug effects.

The microdialysis results are summarized and represented ahead in the main text.

Thus, the long re-exposure (80 min) to the CS+-paired context favored the extinction of the conditioned response. Moreover, it induced a clear-cut increase in DA release in the PL, accompanied by a concomitant decrease in the NAcCo.

Findings from the accompanying preliminary experiments indicate a correlation between the extinction of AMPH-CPP, an increase in DA in the mpFC, and a reduction in DA in the NAc.

These results are consistent with the hypothesis that we present here: that DA transmission in the mpFC and NAc contributes to cognitive/behavioral flexibility by disengaging organisms from behavioral strategies that are ineffective in novel conditions.

## 5. General Discussion, Limitations, and Conclusions

Here, we discuss the novel hypothesis that increased tonic DA levels in the mpFC are required to disengage behavioral responses from species-typical or previously established patterns that are unsuited to a changed environment. This is attained through the inhibitory effects exerted by DA transmission in the mpFC over tonic DA release in the NAc, which reduces the motivational salience of the pursued goal. It is worth pointing out that this mechanism should not work on habit-like responses, which are automatically elicited by the presence of conditioned stimuli and are insensitive to goal devaluation, a behavior that that characterizes compulsion-related psychopathology [13,62].

To support this hypothesis, we report evidence from the literature on dysfunctional cortical–striatal connectivity in neuropsychiatric and neurological disorders, emphasizing the altered DA transmission that characterizes these disorders. Indeed, these data point to a major role of mesocorticolimbic DA transmission. To find evidence of the mechanisms involved, we also discuss findings from animal models on the neuroanatomy of the cortical–subcortical connectivity modulated by DA transmission, the mesocorticolimbic DA responses to unexpected changes in contingencies, and the relationships between these responses and behaviors. These findings point to mpFC catecholamines modulating DA transmission in the NAc in uncertain but relevant situations requiring flexible changes in behavioral strategies. Indeed, in these situations, enhanced NE transmission in the mpFC enhances mesoaccumbens DA to support motivation to escape/avoid negative stimuli or search/pursue positive stimuli. On the other hand, when contingencies change, as in the case of failure to escape/avoid a stressful situation or find/obtain a reward, the reduced availability of released NE and the massive increase in DA in the mpFC are associated with the disappearance of the goal-oriented behavior.

Studies of rewarding or aversive/stressful pharmacological stimuli clearly show that catecholamine transmission in the prefrontal cortex concertedly regulates dopaminergic transmission in the NAc. 

Given the role of the NAc in motivating the acquisition of reward-related CSs, it is conceivable that the reduction in dopaminergic transmission in the NAcCo supports the extinction of reward-related CSs, possibly by reducing the salience of the CS. The reduction in DA in the NAcCo is very likely to depend on increased DA transmission in the mpFC.

Since early studies, the mesocorticolimbic system has been considered to be a regulator of the affective dimension of pain, and attention has been focused on DA transmission from the VTA to forebrain areas including the cortical (mpFC) and subcortical regions (mainly the NAc), without a sharp distinction.

This system was classically considered to be the core of the brain’s reward system, processing the salience and valence of not only rewarding but also aversive stimuli. The close relationship between reward and pain suppression led to their consideration as being on a hedonic continuum, with extreme negative and positive affect located at opposite ends and normal affect located in the middle. This view led some years later to the reward/anti-reward view [64].

Painful stimulation increases DA release in the NAcCo, as suggested by both rodent and human studies, whereas it may suppress dopamine neurotransmission in the NAc shell [75]. Moreover, DA is correlated with the intensity of perceived pain [75], thus indicating that the DA response in the NAc is associated with the emotional response and, thus, with perceived salience.

Our review is not devoid of limitations. A limitation of this review is not having sufficiently considered that the prefrontal/accumbens dopaminergic system is connected through complex neural circuits to other brain systems with which, in some cases, it is reciprocally regulated.

**Figure 2:**
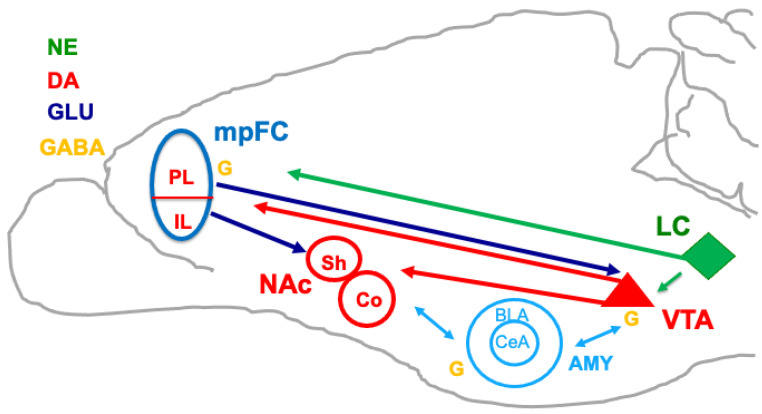
Schematic drawing of the main prefrontal corticolimbic connections possibly involved in the attribution of motivational salience to stimuli, or in salience devaluation involved in extinction. In the present review, we focused on the prelimbic cortex (PL)’s regulation of dopamine in the mesoaccumbens; however, other brain areas and neurotransmitters are likely to play a role (see the limitations in the Discussion section). mpFC, medial prefrontal cortex; PL, prelimbic; IL, infralimbic; NAc, nucleus accumbens; Sh, shell; Co, core; LC, locus coeruleus; VTA, ventral tegmental area; AMY, amygdala; BLA, basolateral; CeA, central nucleus; NE, norepinephrine; DA, dopamine; GLU, glutamate; GABA, γ-aminobutyric acid present in some brain areas, represented by a G close to each area. Sky blue arrows represent reciprocal connections between the amygdala and other brain areas.

The functioning of the nucleus accumbens is to be considered, along with the prefrontal cortex and the amygdala, as a component of the brain circuitry regulating effort-related functions. In this framework, the prefrontal/accumbal CA system may conceivably be a part of a complex network involving cortical and subcortical brain areas involved in the regulation of effort-related functions controlling motivation, and possibly linking salience intensity to effort intensity (Figure 2). In our view, the impact of salient stimuli is crucial in the processes of perceived salience. Thus, the impacts of stimuli produce an emotional response that tunes the association processes leading to the motivation outcome, thus pointing to the basic role of the emotional salience of the unconditioned stimulus to which the individual is exposed. The prefrontal/accumbal system should be considered to be included in complex networks regulating perceived emotion. Emotional perception, according to the appraisal theories (see [27] for a review), stems from three processes: the identification of the emotional significance of a stimulus, the production of an affective state in response to the stimulus, and the regulation of the affective state. These processes depend on different brain emotional systems involving various mesencephalic, cortical, and subcortical areas of the brain, such as the amygdala, insula, ventral striatum, ventral and dorsal anterior cingulate gyrus, and prefrontal cortex, all characterized by reciprocal functional relationships [76]. The amygdala has a well-known role in emotion and in memory consolidation processes depending on emotional arousal, as well as in decision-making. Indeed, the amygdala can evoke conditioned responses capable of exerting a dominant effect on choice, and perceived emotional values in Pavlovian conditioning are exploited by instrumental (i.e., habit-based and goal-directed) learning mechanisms through connectivity with other brain regions, such as the striatum and prefrontal cortex [77]. 

Stress hormones, such as glucocorticoids, through their action on the amygdala, control memory consolidation, thus pointing to a link between emotional salience and the strength of memories [78,79]. Moreover, glucocorticoids are a biological substrate of reward, and they play a role in the modulation of both appetitive and aversive emotional memories [80]. 

However, we have partially compensated for this limitation by inserting the norepinephrinergic system into the dopaminergic system, with specific reference to the role of NE in the mpFC, which exerts a stimulating action on dopaminergic transmission in the NAc, while also forming a prefrontal/accumbens catecholamine system [26]. Therefore, it is a counterpart of prefrontal cortical dopaminergic transmission.

Catecholamine transmission in these regions plays a critical role in different aspects of associative learning, such as consolidation, reconsolidation, and extinction.

Prefrontal NE spurs and DA brakes DA in the accumbens. The prevalence of one cortical catecholamine dampens the action of the other, and as far as cortical dopamine is concerned, an elevated tone lowers the dopaminergic transmission in the accumbens.

Interestingly, increased prefrontal NE outflow occurs in response to both rewarding and aversive unconditioned stimuli (UCSs) with high motivational salience involving the modulation of DA transmission in the NAc [26].

The role of the mpFC’s NE in the accumbal modulation of DA points to the crucial role of the locus coeruleus. The locus coeruleus (LC) is a small collection of noradrenergic neurons located just ventrally and behind the periaqueductal gray in the dorsorostral pons. Although few in number, NE neurons in the LC give rise to highly divergent and extensive projections that release NE in many brain regions to modulate a wide range of behaviors [81].

Several studies have addressed the potential involvement of the LC in the pathogenesis of neurodegenerative disorders [50], and the development of MRI-based methods aimed at visualizing the LC has allowed for the evaluation of its integrity in vivo [49,51]. A crucial role of the LC in pain and stress-related responses has recently been reaffirmed [46].

In PD, the LC is one of the first areas to undergo degeneration. The NE system also exerts anti-inflammatory and neuroprotective effects on DA neurons in the VTA, protecting them from degeneration; consequently, it is highly probable that NE damage in the LC will affect the disease progression (see [82] for a review).

Remarkably, the LC neurodegeneration that characterizes PD and the loss of NE projections are likely to affect pain sensitivity through different mechanisms involving different brain networks, and through the modulation of DA transmission in the NAc. In fact, the LC–VTA connection modulates DA transmission [82,83].

LC degeneration reduces the dopaminergic transmission in the NAc, especially in terms of release, through two mechanisms: by blunting DA stimulation in the NAc, and/or by allowing prefrontal DA to “freely” slow down the dopaminergic transmission in the NAc.

In this way, the activation of the prefrontal/accumbens dopaminergic system, through the transmission in the mpFC, reduces the ability to attribute motivational salience on the one hand, while making the organism vulnerable to aversive and painful stimuli on the other.

This picture clearly indicates that the system must be able to implement flexible and, in some cases, opposite responses in various conditions in which adaptation requires activation (e.g., learning, pain reduction, coping) or a reduction in the transmission at the final point, i.e., the NAc, in those situations in which it is necessary to reduce the goal values.

Moreover, we are aware that the preliminary experiment presented here to support our hypothesis is partial. However, it may be useful as a suggestion for further studies in animal models, as we will comment ahead.

Despite these limitations, we propose our hypothesis of a role of prefrontal/accumbens DA transmission in devaluation, as also supported by the extinction experimental procedure.

Extinction learning has received increased interest in the last two decades. Since it is observed across species, studies on laboratory animals such as rodents have allowed researchers to investigate the underlying biological substrate. Moreover, extinction learning is of huge clinical relevance, since it is the basis of exposure therapy. Therefore, the optimization of extinction learning is a subject of intense investigation [84].

When learned associations are recalled from long-term memory stores as a result of the presentation of an unreinforced CS, extinction, through presentation of the CS alone, degrades the association between the CS and UCS. Interestingly, conditioning and extinction have been reported to induce opposing changes in synaptic connections in the cortex [85].

Modulation of the motivational properties favors disengagement from cues related to UCSs. Thus, therapy strategies are aimed at reducing the motivational properties of UCS-related cues.

Indeed, in psychotherapy, exposure techniques are based on extinction procedures. Extinction also involves the attribution of motivational salience and has a crucial neural basis in the prefrontal/accumbens dopaminergic system.

Evidence shows that the NAcCo subregion plays a crucial role in the extinction of rewarding or aversive CSs, and that the extinction of CSs to rewarding UCSs leads selectively to relevant neuroplasticity in the NAcCo, as expressed by increased BDNF levels [71]. Remarkably, DA in the NAcCo is moderated by the prelimbic portion of the mpFC and the PL, and the mpFC’s cortical DA plays a major role in cognitive flexibility and in the individual adaptive responses to changes occurring in the external environment, as well as being crucial in the extinction of aversive and appetitive experiences.

A methodological approach in an animal model capable of investigating learning and extinction in parallel with DA release in the mpFC and the NAc allows us to verify this hypothesis. Thus, we carried out a CPP experiment in rats to assess in vivo dopamine release concomitantly in the mpFC and in the NAcCo by intracerebral microdialysis.

**Figure 3:**
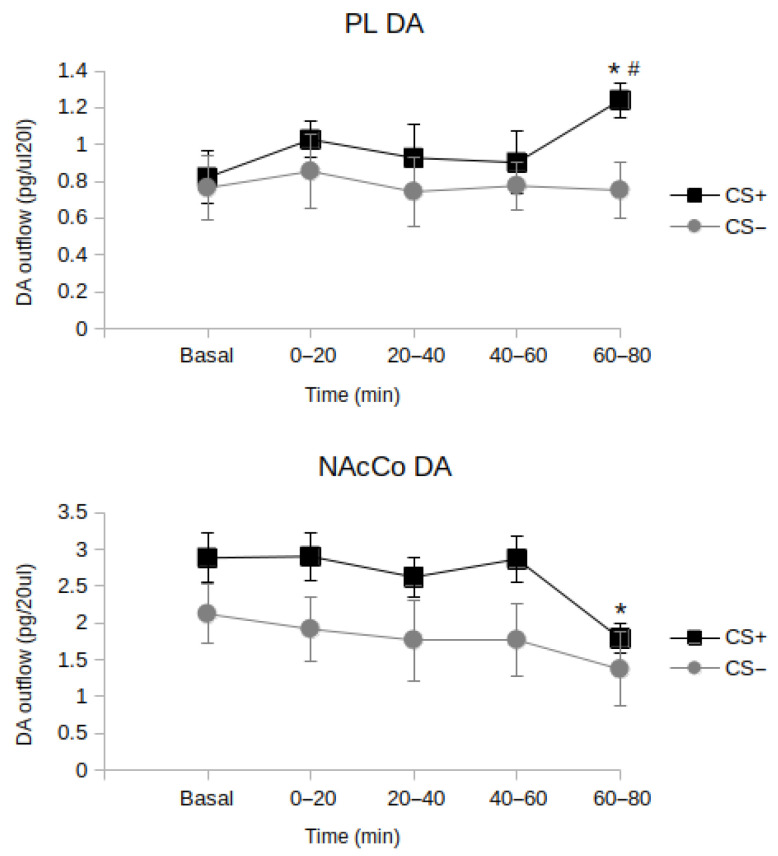
Effects of 80 min of re-exposure to CS+ or CS− on DA outflow in the *prelimbic prefrontal cortex* (PL) and *nucleus accumbens core* (NAcCo). CS+ = chamber associated with AMPH. CS− = chamber associated with saline. Results are expressed as pg/20 µL concentration (means ± SE); * *p* < 0.05 in comparison with basal levels, # *p* < 0.01 in comparison with CS− in the corresponding time block.

The results showed that the long (80 min) but not short (up to 60 min) re-exposure to the US-paired context (CS+) favored the extinction of the conditioned response. Moreover, the long re-exposure to CS+ caused a significant increase in the DA release in the PL cortex in the last time block (60–80 min) of re-exposure. In contrast, in the same time block, DA in the NAcCo dropped drastically (Figure 3). These findings point clearly to a crucial role of DA in the PL in enhancing the acquisition and maintenance of the extinction. Although the experiment did not demonstrate a cause–effect relationship between the increase in DA in the PL and the decrease in DA in the NAcCo, consolidated data in the literature strongly suggest that this is the case. 

Moreover, although not even a causal relationship was demonstrated between the prefrontal/accumbal DA system response and extinction, it is very likely that the clear decrease in DA in the NAcCo supports extinction, possibly by reducing the salience of the CS, due to the reduction in DA in the NAcCo caused by increased DA transmission in the PL. Our preliminary results confirm the hypothesis discussed here, that increased tonic DA levels in the mpFC are required to disengage behavioral responses from previously established behavioral patterns that do not fit with a changed environment. This is attained via the inhibitory effects exerted by DA transmission in the mpFC over tonic DA release in the NAc, which reduces the motivational salience of the pursued goal.

Both behavioral and neurochemical results are relevant, since they point, for the first time to our knowledge, to the potential therapeutic power of the prefrontal/accumbal DA system. Indeed, research should deepen the knowledge of the molecular mechanisms involved in the regulation of the prefrontal/accumbal dopaminergic system and its role in behavioral flexibility [86]. New knowledge could lead to the development of pharmacological tools to be combined with psychotherapy to produce a therapeutic synergy that proves to be increasingly effective.

Chemogenetics and optogenetics studies [87,88] will possibly ascertain the causal relationship between PL and NAcCo DA transmission in the devaluation of salient learned associations. Indeed, by silencing or enhancing neurotransmission in the PL, the effects on neural activity in the NAcCo will unveil its involvement in perceiving the salience of conditioned stimuli. The involvement of other neurotransmitters in brain networks in which the PL and NAcCo are included, such as corticostriatal glutamatergic transmission or norepinephrine’s modulation of accumbal DA transmission, to name but a few, may point to molecular factors that could be relevant for pharmacological therapy. Indeed, human and animal model research, through imaging and molecular analysis methods, can converge to facilitate the discovery of molecular and neurobehavioral biomarkers, to understand pathogenic mechanisms, and to develop effective therapies.

Pharmacological and psychological therapies are notoriously allies in the treatment of neurobehavioral disorders. Although they are extremely useful, the drugs are mainly administered systemically and, therefore, cannot reach specific brain areas in a selective and exclusive way.

In the case of the PL–NAcCo system, the possibility of acting directly and specifically on the frontal cortical dopaminergic transmission would offer the prospect of a relevant tool for modulating the transmission in the cortex and the regulation of the subcortical regions, showing promise in treating a range of psychiatric and neurological conditions.

Recently, transcranial direct-current stimulation (tDCS), an electrostimulation technique that is considered to be non-invasive, has been used with the aim of cortical and cortical–subcortical modulation in animals and humans, with therapeutic and scientific investigation purposes. In fact, tDCS has been shown to specifically stimulate dopaminergic transmission in the frontal cortex, and its combination with neuroimaging analysis has proven to be of huge utility [89].

tDCS DA stimulation of the frontal pole has been reported to affect the substantia nigra and ameliorate motor deficits in PD patients [90]. Moreover, tDCS has been recently reported to affect decision-making [91] in cognitive flexibility [92,93] or attention [94], with improving effects.

**Figure 4:**
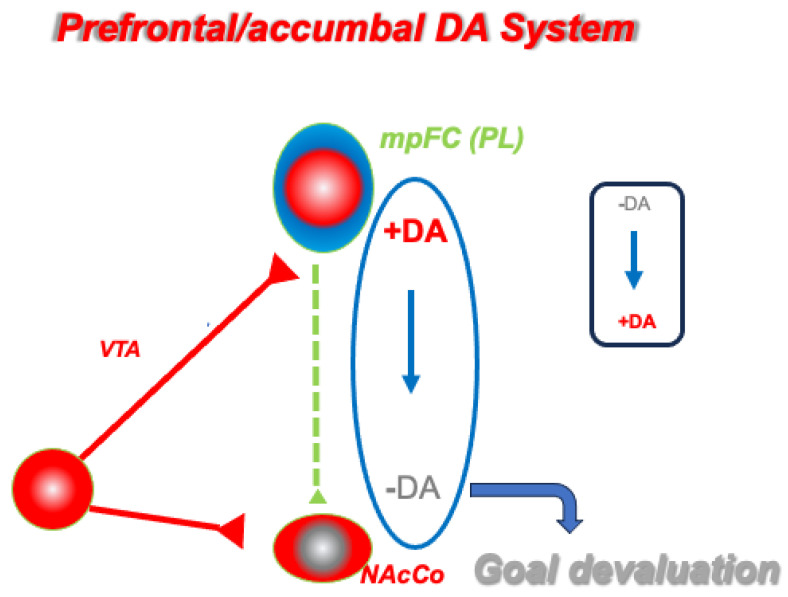
Schematic representation of our hypothesis of prefrontal (PL) dopamine (DA) modulation of DA transmission in the nucleus accumbens core (NAcCo). An inverse relationship between DA transmission in the PL and NAcCo modulates the goal values. High (+) DA in the PL causes a decrease (−) in DA in the NAcCo and a goal devaluation (low DA and goal devaluation in grey). The small rectangle shows the inverse relationship.

These research and therapy perspectives are consistent with the hypothesis that we present here, as summarized in Figure 4, that DA transmission in the mpFC and NAc contributes to cognitive/behavioral flexibility by disengaging organisms from behavioral strategies that are maladaptive in changing conditions.

## Supplementary Materials

The following supporting information can be downloaded at: https://www.mdpi.com/article/10.3390/biomedicines11123189/s1, Refs [71,95,96,97,98].

## Abbreviations

CS: conditioned stimulus; US: unconditioned stimulus; DA: dopamine; VTA: ventral tegmental area; SN: substantia nigra; SNpc: substantia nigra pars compacta; mpFC: medial prefrontal cortex; PL: prelimbic cortex; NAc; nucleus accumbens; NAcCo: nucleus accumbens core; NE: norepinephrine; D2R: DAD2 receptor; PD: Parkinson’s Disease; ACC: anterior cingulate cortex; LC: locus coeruleus; CPP: conditioned place preference; AMPH: amphetamine; tDCS: transcranial direct-current stimulation; UPLC: ultra-performance liquid chromatography; ***Motivational salience***: A mental process that motivates or propels an individual’s behavior towards or away from a particular object, perceived event, or outcome. ***Reversal learning***: A task aimed at measuring the ability of an experimental subject to inhibit a previously acquired behavioral response and acquire a different or even an opposite one. ***Stress coping***: The behavioral strategies adopted to deal with a stressful situation.
