# Supplementary material for: Prefrontal Dopamine in Flexible Adaptation to Environmental Changes: A Game for Two Players
Source: Biomedicines. 2023 Nov 30;11(12):3189. doi: 10.3390/biomedicines11123189 (PMC10740496; doi:10.3390/biomedicines11123189)
Supplement: Supplementary file 1 [file biomedicines-11-03189-s001.zip › biomedicines-2661020-supplementary.pdf]

SUPPLEMENTARY MATERIALS

In the present study, we used a paradigm of spatial conditioned preference (CPP) induced by amphetamine (AMPH) in rats to assess the effects of a short re-exposure (20 minutes) or a long re- exposure (80 minutes) to the drug-paired context on the extinction of the conditioned drug- seeking response. Moreover, given the critical role of dopamine in mpFC (prelimbic cortex, PL) and nucleus accumbens (Core) in the extinction of conditioned, we assessed the time-course of DA in these areas during the re-exposure to the drug-conditioned context using in vivo microdialysis.

MATERIALS and METHODS

>Animals and Housing. Male Sprague-Dawley rats (Charles River, Lecco, Italy), weighting 300-400 gr at the beginning of the experiments, were housed individually and maintained in a 12 h/12 h light-dark cycle (lights on between 7.00 a.m. and 7.00 p.m.). Rats had free access to standard laboratory chow and water throughout the entire experiment. Each experimental group consisted of 6-8 animals. All experiments were carried out in accordance with Italian national law (DL 116/92 and DL 26/2014) on the use of animals for research based on the European Communities Council Directives (86/609/EEC and 2010/63/UE) and approved by the ethics committee of the Italian Ministry of Health (license/approval ID #: 10/2011-B and 42/2015-PR).
Drugs. D-Amphetamine (AMPH) sulphate was purchased from Sigma (Sigma Aldrich, Milano, Italy). AMPH (2.5 mg/kg) was dissolved in saline (0.9 % NaCl) and injected intraperitoneally (i.p.) in a volume of 1 ml/kg. Zoletil 100 (Virbac, MI, Italy) (Tiletamine HCl 50 mg/ml + Zolazepam HCl 50 mg/ml) and Rompun 20 (Bayer S.p.A, MI, Italy) (Xylazine 20 mg/ml), purchased commercially, were used as anaesthetics.

Behavior. Behavioral experiments were performed using a conditioned place preference (CPP) apparatus [95, 96]. The apparatus comprised two grey Plexiglas chambers (30×30×30 cm) and a central alley (30×15×30 cm). Two sliding doors (15×30 cm) connected the alley to the chambers. In each chamber, two triangular parallelepipeds (10×10×30 cm) made of black Plexiglas and arranged in different patterns (always covering the surface of the chamber) were used as conditioned stimuli (CS) **(Figure S1, S3).** Behavioral data were collected and analyzed by “EthoVision” (Noldus Information Technology, Wageningen, The Netherlands), a fully automated video tracking system. The acquired digital signal was then processed by the software to extract the time spent in second (s) in the three chambers of the apparatus.
On day 1 (pre-test), rats were free to explore the entire apparatus for 20 min. On the following 6 days (conditioning phase), rats were injected and confined daily for 40 min alternatively in one of the two chambers. One of the patterns was consistently paired with saline injections and the other one with amphetamine injections. Pairings were balanced so that for half of the animals in each experimental group, AMPH was paired with one of the patterns and half of them with the other one. The dose of AMPH was chosen based on a previous study [95, 96], and preliminary experiments. Testing was conducted on day 8 in a drug-free state and lasted 20 min, as in pre-test. On days 9 and 10, animals were re- exposed to the drug-paired chamber (day 9) or the saline-paired chamber (day 10) for 20 minutes (20 minutes group, n=6) or 80 minutes (80 minutes group, n=8). On the following 7 days, to investigate potential differences in the

extinction between two groups, animals were exposed daily to CPP testing (20 min) (non-confined extinction). The extinction of the conditioned response was considered acquired after two consecutive days with no significant preference for the drug-paired chamber [96, 98].

Surgery. Additional groups were conditioned for the microdialysis experiment. The day following CPP test animals were subjected to surgical procedures. Rats, anesthetized with Zoletil 100 and Rompun 20, were mounted in a stereotaxic frame (David Kopf Instruments, Tujunga, CA). An incision was performed along the midline of the skull, then a vertical concentric dialysis probes were prepared with AN69 fibers (Hospal Dasco, Bologna, Italy and implanted vertically at the level of the PL cortex (AP +3.7; ML ±0.5; DV -5) or nucleus accumbens core (NAcCo) (AP +2.2; ML ±1.7; DV -8.5), compared to bregma, according to the atlas of Paxinos and Franklin (The Mouse Brain in Stereotaxic Coordinates, 2nd Edn. San Diego, CA: Academic Press.2001) [97] **(Figure S2).** Microdialysis probes were implanted unilaterally 24 h before experiments, secured with dental cement and epoxy resin. Then, the skin was sutured, rats were returned to their home cages and the outlet and inlet probe tubing were protected by locally applied PE-20 tubing. Membranes were tested for in vitro recovery of DA the day before surgery.

Microdialysis. On the day of the experiment, each animal was placed in the CPP apparatus containing microdialysis equipment: the microdialysis probe was connected to a CMA/100 pump (Carnegie Medicine) through PE-20 tubing and an ultra-low torque multichannel power- assisted swivel (Model MCS5, Instech Laboratories) to allow free movement. Artificial Cerebral Spinal Fluid (CSF; 147 mM NaCl, 1mM MgCl, 1.2 mM $CaCl_2$, and 4 mM KCl) was pumped through the dialysis probe at a constant flow rate of 2.1 µl/min. Experiments were carried out 22-24 h after probe placement. At the start of the dialysis perfusion, rats were confined in the central alley, and left undisturbed for about 1 h before the collection of three baseline samples. The mean concentration of the three samples collected was taken as basal levels concentration (basal). After the third sample, rats were confined in the drug-paired chamber or saline-paired chamber. Four dialysate samples were collected every 20 min for a total of 80 minutes of re-exposure. Only data from rats with a correctly placed probe are reported.

Monoamine analysis. Twenty microliters of each dialysate sample were analyzed by Ultra-Performance Liquid Chromatography (UPLC). The UPLC system consisted of an ACQUITY UPLC (Waters Corporation, Milford, MA) apparatus coupled to an amperometric detector (Model Decade II, Antec Leyden, The Netherlands) equipped by an electrochemical flow cell (VT-03, Antec Leyden) with 0.7 mm glassy carbon working electrode, mounted with a 25 mm spacer and an *in situ* Ag/AgCl (ISAAC) reference electrode. The electrochemical flow cell was placed immediately after a BEH C18 column (2.1×50 mm, 1.7 µm particle size; Waters Corporation) and set at 400 mV of potential. The column was maintained at 37°C and the flow rate was 0.07 ml/min. The mobile phase was composed of 50 mM phosphoric acid, 8 mM KCl, 0.1 mM EDTA, 2.5 mM 1-octanesulfonic acid sodium salt 12% MeOH and pH 6.0 adjusted with NaOH. Peak height produced by oxidation of DA was compared with that produced by the standard. The detection limit of the assay was 0.1 pg [99].
At the end of the experiments, rats were killed. Brains were post-fixed in 4% paraformaldehyde and correct probe placements were checked by visual inspection of the probe tracks on Nissl-stained coronal sections (40 µm). Only rats with correct probe placement were considered in the results.

Statistics CPP. Statistical analysis was performed referring to the time spent (s) within each chamber: center, paired and unpaired. The time spent was measured in pre-test, test and

extinction trials. Conditioned place preference was analyzed subtracting the time spent in each chamber (center, paired, unpaired) during pre-test at the time spent in corresponding chambers during test and extinction sessions. The values were subsequently analyzed by one-way ANOVA and revealed non-significant differences in time spent in the two lateral chambers, showing that the apparatus was unbiased in terms of preferences.
Data were analyzed by repeated measures ANOVA with one between factor (group, two levels: 20 minutes, 80 minutes) and one within factor (choice, three levels: center, paired, unpaired). Post hoc comparisons were performed by Duncan's multiple-range test and t-test whenever significant interaction or main effects were attained.

Statistics microdialysis. Statistical analyses were performed on raw data (concentrations, pg/20µl) by two-way ANOVA with one between factor [CS exposure, two levels: CS+ (AMPH-paired chamber) or CS- (Sal-paired chamber)] and one within factor (time, five levels: basal, 0-20, 20-40, 40-60, 60-80). Differences in DA outflow (within factors) were analyzed by a t-test (paired) or one-way ANOVA.

RESULTS

Effects of 20 or 80 minutes of CS re-exposure on extinction of AMPH-induced CPP.
Two-way ANOVA revealed non-significant effect (ns) for group × choice during pre-test [$F(2,24)=0.169$, ns]. All groups did not show significant differences between time spent in the two lateral chambers. Following conditioning all rats expressed a preference for the previously AMPH-paired chamber. Two-way ANOVA revealed a ns interaction choice × group [Test, $F(2,24)=0.091$, ns]. Two-way ANOVA revealed significant effect for the factor choice [Test, $F(2,24)=6.850$, $p<0.0044$]. Duncan's post-hoc analyses showed that all groups spent more time in the amphetamine-paired chamber during CPP Test ($p<0.01$).
Following CS re-exposures, the 20 minutes group showed significative place preference for the AMPH-paired chamber on extinction trials 1 ($p<0.01$), 2 ($p<0.01$), 3 ($p<0.05$), 4 ($p<0.05$). This group reached the extinction criterion of two consecutive days with non-significant preference on days 5-6, showing again preference on day 7 ($p<0.01$). The 80 minutes group showed significative place preference for the AMPH-paired chamber on extinction trials 1 ($p<0.01$) and 2 ($p<0.01$), reaching the extinction criterion on extinction trials 3-4 and maintaining extinction till the end of experiments **(Figure S4).**
Results show that a long re-exposure (80 min) to the drug-paired chamber favours acquisition and maintenance of extinction. Indeed, the 80 min group reached the extinction criterion (two consecutive trials with non-significant preference) on days 3-4, maintaining the CR inhibition for the entire experiment (till day 7), while animals re-exposed for 20 minutes reached extinction criterion significantly later, at days 5-6.

Effects of 80 minutes re-exposure to CS+ on DA outflow.

During pre-test, all groups showed no significant differences between time spent in the two lateral chambers. Following conditioning, during CPP Test, all animals showed a preference for the chamber previously paired with AMPH [Test, $F(2,18)=4.120$, $p<0.001$].
The effects of 80 minutes of CS re-exposure on DA outflow in PL cortex are reported in Figure 1. Re-exposure to the paired chamber did not induce significant changes vs baseline till 60 minutes, while a clear-cut significant increase was evident in the last time block (60-80 m.) ($p< 0.01$, t-test paired).
Eighty minutes CS re-exposure showed no significant differences in NAcCo DA outflow from the

baseline, both in CS+ and CS- re-exposed groups in the 0-20, 2-40- 40-60 intervals, while DA levels decreased significantly at the 60-80 interval in CS+ group. Two-way ANOVA revealed a non-significant interaction at time × CS [$F(1,48)=1.109$, ns], and at CS factor [$F(1,48)=2.528$, ns]. However, it showed a significant effect of time on DA decreased release [Time, $F(4,48)=7.525$, $p=0.0001$]. Indeed, t-test showed significant difference between the last time block (60-80 m.) of exposure to CS+ and the corresponding basal level (mean diff 1,097; t-test=2,669, $p<0.02$).

The present results show that 60 minutes CS+ re-exposure did not induce significant DA changes in PL. However, in the final time block of CS+ re-exposure (60-80 min), DA levels increased dramatically as shown by significant difference from baseline levels and from DA release induced by exposure to CS- at the corresponding time block (60-80 min).
Re-exposure to CS+ produced in NAcCo a DA release time-course that remained stable at basal levels for about 60 minutes and then decreased significantly at the 60-80 interval.
Thus, the long re-exposure (80 min) to the CS+ paired context favours extinction of the conditioned response. Moreover, it induced a clear-cut increase of DA release in PL accompanied by a concomitant decrease in the NAcCo.
These findings strongly suggest a crucial role of DA outflow in PL and NAcCo in enhancing the acquisition and maintenance of the extinction. Although this preliminary experiment does not demonstrate a cause-effect relationship between the increase in DA in PL and the decrease in DA in the NAcCo, consolidated data in the literature strongly suggest that this is the case. Thus, it is conceivable that the reduction of dopaminergic transmission in NAcCo supports extinction, possibly by reducing the salience of the CS.

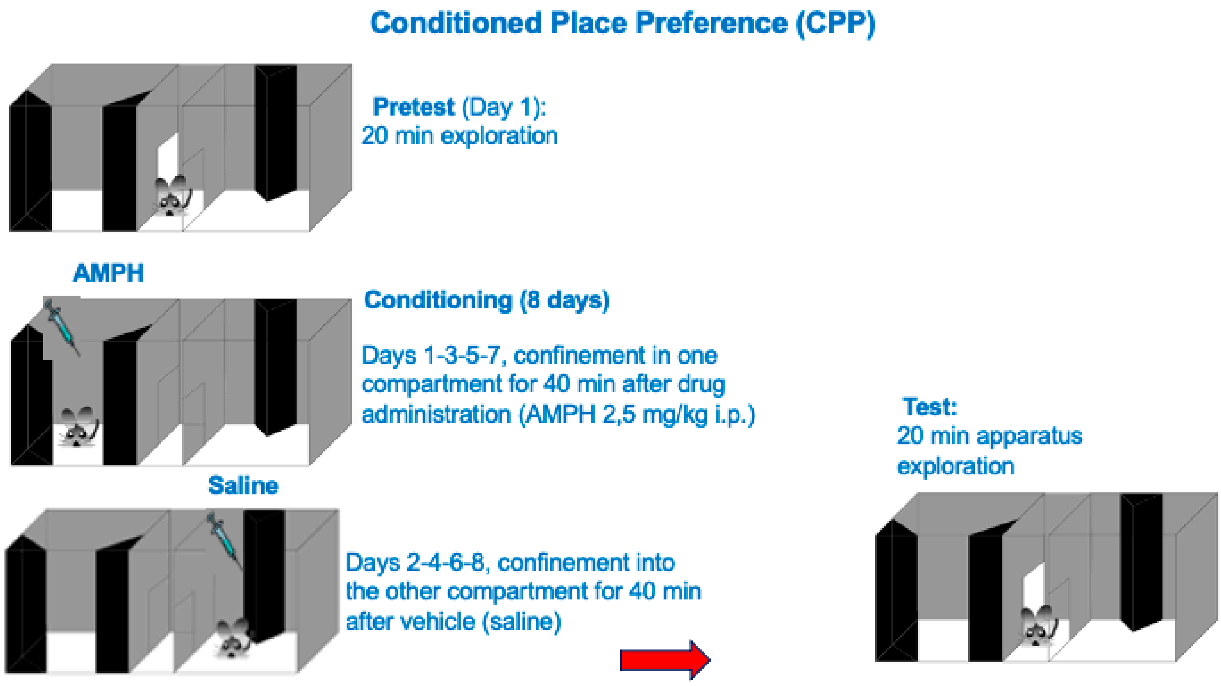

Figure S1. Conditioned Place Preference apparatus and conditioning procedure.

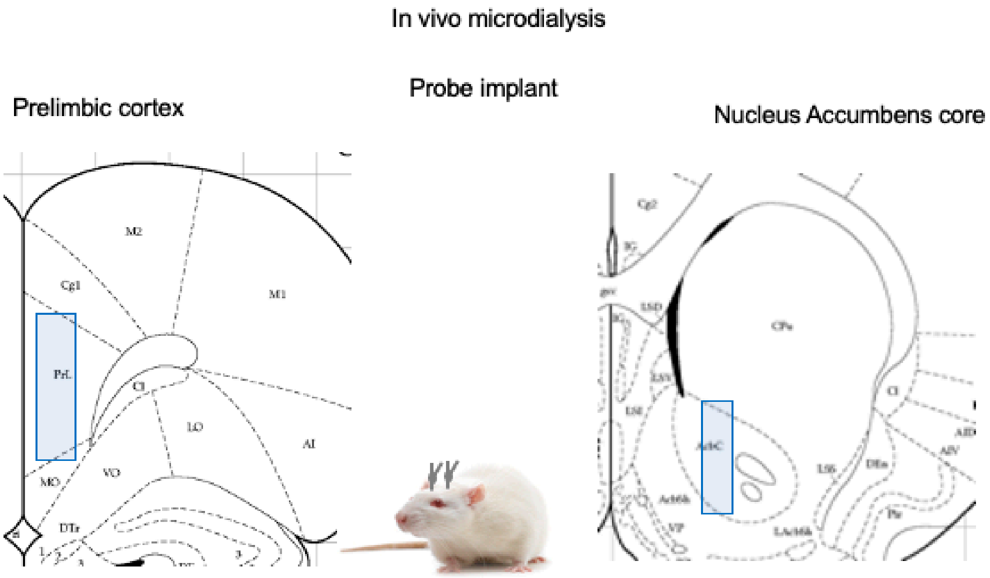

Figure S2. Location of microdialysis probes in the Prelimbic cortex and in the Nucleus accumbens core of rat brain.
The inset represents the areas containing the tracks of the microdialysis probes and the range of implantation sites. Representative sections of the rat brain adapted from the atlas Paxinos and Watson (1998). For details, see "Materials and methods"

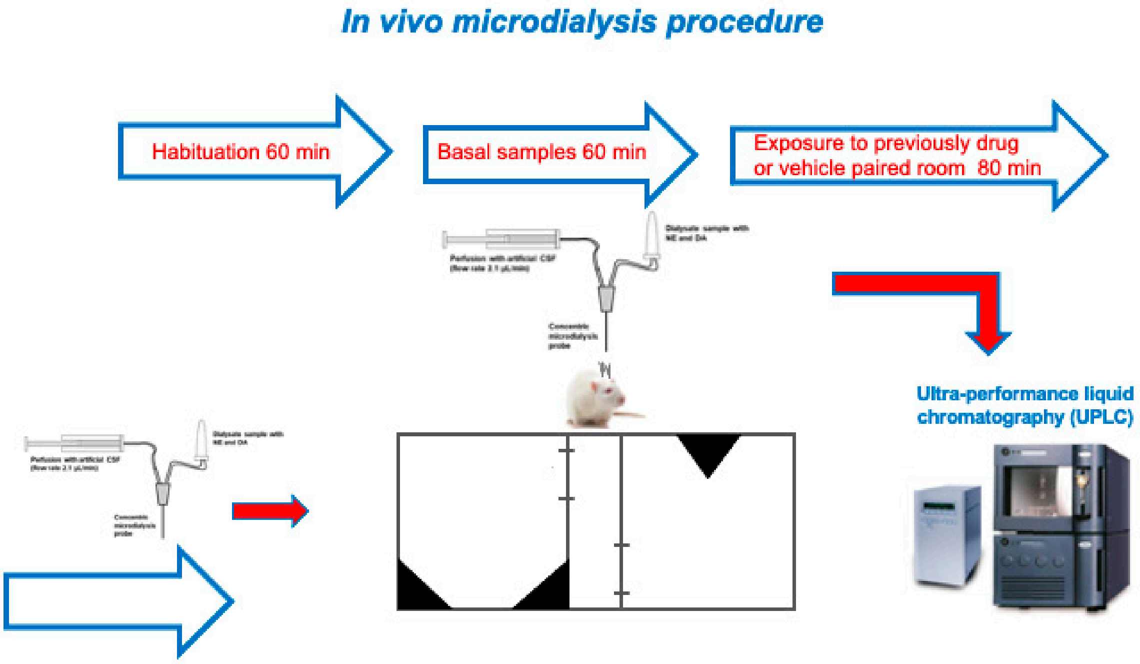

Figure S3. Microdialysis and behavioral set.
Microdialysis procedure is represented with Conditioned Place Preference apparatus, dialysate sample collection, and UPLC analysis of dopamine idyalisate.
Details are in Material and Methods.

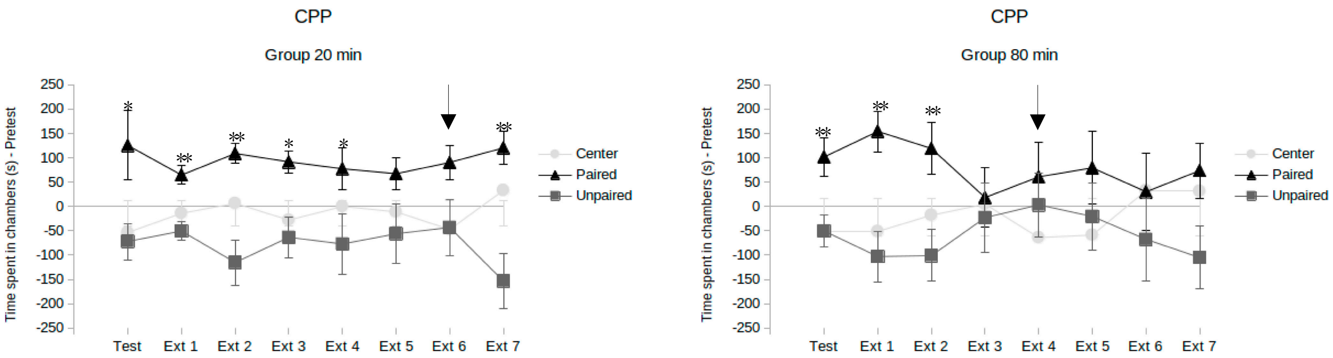

Figure S4. **Effects of 20 or 80 minutes of CS re-exposure on the extinction of AMPH-induced CPP.**

All data are expressed as mean (second±SE) time spent in Center, Paired and Unpaired chambers during Test (ordinate) or Extinction (Ext) trials (abscissa). *p<0.05, **p<0.01 in time spent in paired comparison with unpaired chamber. Arrows indicate the achievement of the extinction criterion of two consecutive days of no-preference. Details are in Materials and Methods.
